# DNA methylation directs microRNA biogenesis in mammalian cells

**DOI:** 10.1038/s41467-019-13527-1

**Published:** 2019-12-11

**Authors:** Ohad Glaich, Shivang Parikh, Rachel E. Bell, Keren Mekahel, Maya Donyo, Yodfat Leader, Ronna Shayevitch, Danna Sheinboim, Sivan Yannai, Dror Hollander, Ze’ev Melamed, Galit Lev-Maor, Gil Ast, Carmit Levy

**Affiliations:** 1 Department of Human Genetics and Biochemistry, Sackler Faculty of Medicine, Tel Aviv University, 69978 Tel Aviv, Israel; 2 Present Address: Ludwig Institute for Cancer Research and Department of Cellular and Molecular Medicine, University of California at San Diego, La Jolla, CA 92093 USA

**Keywords:** DNA methylation, miRNAs

## Abstract

MicroRNA (miRNA) biogenesis initiates co-transcriptionally, but how the Microprocessor machinery pinpoints the locations of short precursor miRNA sequences within long flanking regions of the transcript is not known. Here we show that miRNA biogenesis depends on DNA methylation. When the regions flanking the miRNA coding sequence are highly methylated, the miRNAs are more highly expressed, have greater sequence conservation, and are more likely to drive cancer-related phenotypes than miRNAs encoded by unmethylated loci. We show that the removal of DNA methylation from miRNA loci leads to their downregulation. Further, we found that MeCP2 binding to methylated miRNA loci halts RNA polymerase II elongation, leading to enhanced processing of the primary miRNA by Drosha. Taken together, our data reveal that DNA methylation directly affects miRNA biogenesis.

## Abstract (web summary)

Long primary transcripts of microRNAs are co-transcriptionally cleaved by the enzyme Drosha. Here the authors suggest that DNA methylation in miRNA loci in mammalian cells increases Drosha binding, slowing RNA polymerase II elongation to enhance miRNA biogenesis.

## Introduction

MicroRNAs (miRNAs) are noncoding RNAs of about 22 nucleotides in length that play important roles in regulation of gene expression and thus in various developmental and pathological processes^1,2^. miRNAs can be either intergenic or intronic and are located in both coding and noncoding genes^3,4^. miRNAs are usually transcribed by RNA polymerase II (Pol II) as part of longer primary transcript known as the pri-miRNA^5^. Cleavage of pri-miRNAs occurs co-transcriptionally and is catalyzed by the RNase III enzyme Drosha. Drosha and its co-factor DGCR8 are part of the Microprocessor complex, which is involved in the early stages of processing microRNA^3,4^. Cleavage of the pri-miRNA produces the precursor miRNA hairpin (pre-miRNA), which is exported to the cytoplasm where it is cleaved by Dicer to generate the mature miRNA^6^. The mature miRNA strand is then preferentially incorporated, together with the Argonaute protein AGO2, into the RNA-induced silencing complex or RISC^3,4^.

DNA methylation, nucleosome positioning, and Pol II elongation rate are known to influence messenger RNA biogenesis^7–16^, and we reasoned that these factors might also regulate miRNA biogenesis. DNA methylation is an epigenetic marker found predominantly on cytosine in CpG dinucleotides that is written by DNA methyltransferases (DNMTs)^17^. DNA methylation is directly involved in regulation of gene expression through impacts on promoter or enhancer accessibility^18,19^. For instance, hypermethylation of promoters results in gene silencing^20^. DNA methylation is dynamically remodeled during the mammalian life cycle through distinct phases of reprogramming and de novo methylation^21^. A known reader of DNA CpG methylation is the 5-methylcytosine-binding protein MECP2^22,23^. MECP2 is involved in transcription regulation, miRNA processing, and precursor messenger RNA (mRNA) splicing^24–27^.

DNA methylation is known to influence the splicing machinery^25,28–32^. Exon sequences tend to have higher methylation levels than the flanking intron sequences, and constitutively spliced exons have higher methylation levels than alternatively spliced exons^30^. Exons also have higher GC content than introns, and GC content is directly correlated with DNA methylation levels^33,34^. Moreover, DNA methylation influences alternative splicing by mediating the binding of several proteins^25^. One of these proteins is MeCP2, which binds to methylated DNA to promote a kinetic coupling between the elongation rate of Pol II and splicing^29^.

Interestingly, in miRNA loci that have promoters with high levels of CpG dinucleotides, alteration of methylation decreases levels of miRNA expression^35–38^. Moreover, methylation levels over miRNA genomic regions are significantly higher than methylation levels over protein coding genes^39^, and the proportion of miRNAs that reside directly inside CpG islands is higher than expected^40^. DNA methylation in the miRNA gene body suggests that DNA methylation is directly involved in the regulation of miRNA biogenesis; here we interrogate this hypothesis.

Pol II is a major regulator of various RNA-processing events including splicing, capping, and polyadenylation of mRNAs^41,42^. The C-terminal domain (CTD) of the largest subunit of Pol II is differentially phosphorylated during transcription. In the form of Pol II that initiates transcription, the CTD is phosphorylated on Ser5 (pSer5), whereas during elongation, the Ser5 phosphates are removed and the CTD is phosphorylated on Ser2 (pSer2)^43,44^. The existence of phosphorylation at Ser5 without pSer2 implies Pol II pausing^45^. It has been shown that if Pol II is bound on exons it is predominantly phosphorylated at Ser5^46^. This suggests that this modification may induce a pause in transcription to allow the nascent exon to be recognized by the splicing machinery^45^.

In the analyses described here, we found that miRNAs encoded by loci in which the regions flanking the pre-miRNA coding sequence are highly methylated are characterized by significantly higher levels of mature miRNA expression and higher evolutionary conservation compared to the loci that are not methylated or that do not have differential methylation. We further found that biogenesis of the miRNAs encoded by the highly methylated DNA is more perturbed upon changes in methylation than is biogenesis of those miRNAs encoded by unmethylated DNA. miRNAs from methylated loci are significantly downregulated in response to methylation removal and also have a lower Drosha occupancy over their genomic regions compared to those from unmethylated loci. We also found that the rate of Pol II elongation is slower across methylated miRNA loci than unmethylated loci and that the increased Pol II elongation rate results in reductions in mature miRNA levels. We discovered that MeCP2 binds to methylated CpG to act as a roadblock to Pol II elongation to enhance miRNAs biogenesis. Finally, we found that methylated miRNAs are significantly enriched with cancerous phenotypes compared to unmethylated miRNAs.

## Results

### DNA methylation marks the locations of pre-miRNA boundaries

**Fig. 1:**
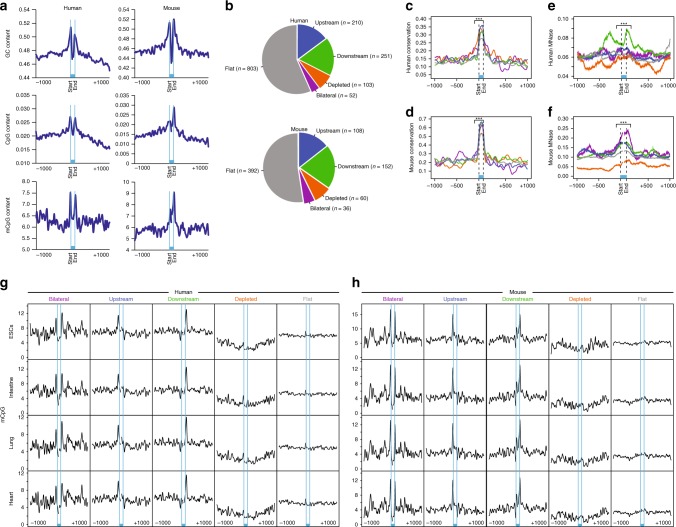
Methylation marks the locations of pre-miRNA boundaries at the DNA level. **a** GC content (upper panel), CpG content (middle panel), and methylated CpG content (lower panel) across 100 bp of miRNA-encoding sequence and an additional 1000 bp of the flanking regions for human and mouse. The miRNA location is indicated in light blue. **b** Pie charts illustrating the different methylation profiles found in human and mouse miRNA loci. **c**, **d** Plot of mean conservation scores across 100 bp of miRNA and additional 1000 bp of the flanking regions for **c** human and **d** mouse. To evaluate statistical significance the miRNA regions (100 bp) were tested against 100 bp of adjacent upstream regions. All *t*-tests were significant with ***p < 0.0001. **e**, **f** Plots show MNase-seq signal reflective of nucleosome occupancy across 100 bp of miRNA and additional 1000 bp of the flanking regions in (**e**) humans and (**f**) mouse. To evaluate statistical significance, miRNA regions (100 bp and an additional 50 bp of the adjacent upstream and downstream regions) of bilateral, upstream, and downstream groups were tested against depleted and flat groups. All *t*-tests were significant with ***p < 0.0001. **g**, **h** miRNA methylation profiles in (**g**) human and (**h**) mouse intestine, lung, heart, and embryonic stem cells.

To test if DNA methylation affects miRNA biogenesis, we first analyzed the GC content (the proportion of guanine and cytosine nucleotides relative to the total number of nucleotides) of all pre-miRNA loci in the human and mouse genomes. Pre-miRNA loci were scaled to a length of 100 base pairs (bp) and a mean GC level was determined for each base pair. Pre-miRNA coding regions have higher GC content compared to the adjacent upstream and downstream regions (Fig. 1a, top panel). We repeated this analysis with the CpG dinucleotide content (the proportion of CpG dinucleotides relative to the total number of dinucleotides) and observed the same result (Fig. 1a, middle panel). The higher frequency of CpG dinucleotides offers a higher chance for CpG methylation to occur in these regions.

Next, we examined the DNA methylation levels of miRNA loci and their flanking sequences. We analyzed whole-genome bisulfite sequencing data for human H1 embryonic stem cells (ESCs)^47^ and mouse R1 ESCs^48^ obtained from the ENCODE project. Pre-miRNAs had higher levels of CpG methylation compared to their flanking regions, and methylation levels were especially high at the boundaries of pre-miRNAs in both human and mouse (Fig. 1a, bottom panel). The overall high level of DNA methylation in pre-miRNA regions suggests that DNA methylation might be important for the biogenesis of certain miRNAs.

By analysis of methylation levels of the pre-miRNA loci in both human and mouse, we identified five distinct patterns (Fig. 1b and Supplementary Data 1): methylation at both edges of the pre-miRNA (bilateral), methylation only at the 5ʹ (upstream) or 3ʹ (downstream) edge, and no methylation at pre-miRNA borders resulting from either depletion (depleted) or flattening (flat). These five miRNA groups represent 93% of all known miRNA loci of mouse and human; 7% of miRNA loci had ambiguous methylation profiles. The pre-miRNA sequences in these five groups are significantly more evolutionary conserved than their flanking sequences in both human (Fig. 1c) and mouse (Fig. 1d), and the methylated pre-miRNA loci (bilateral, upstream, and downstream; hereafter referred to as methylated miRNAs) are more evolutionary conserved than the unmethylated pre-miRNA loci (depleted and flat; hereafter referred to as unmethylated miRNAs) (Fig. 1c, d). Nucleosome position is highly correlated with DNA methylation pattern throughout the genome as DNA methyltransferases preferentially target nucleosome-bound DNA^49^. We reasoned that higher nucleosome occupancy might serve as an additional epigenetic marker for miRNA biogenesis. In support of this hypothesis, nucleosome occupancy was significantly higher over loci of methylated miRNAs than unmethylated miRNAs in both human (Fig. 1e) and mouse (Fig. 1f) ESCs.

We found that the methylation patterns of these five groups are the same as those observed in ESCs in various tissues from both human and mouse, including intestine, lung, heart, skin, liver, kidney, and brain as shown by the ENCODE project (see methods) (Fig. 1g, h and Supplementary Fig. 1). This indicates that methylation patterns of miRNA loci are conserved from the embryonic to the differentiated stage in a species-specific manner and across the various cell types in an organism. In addition, the CpG content was mostly correlated with methylation pattern (Supplementary Fig. 2). Altogether, these findings strengthen our hypothesis that DNA methylation regulates miRNA expression.

### DNA methylation affects miRNA biogenesis

**Fig. 2:**
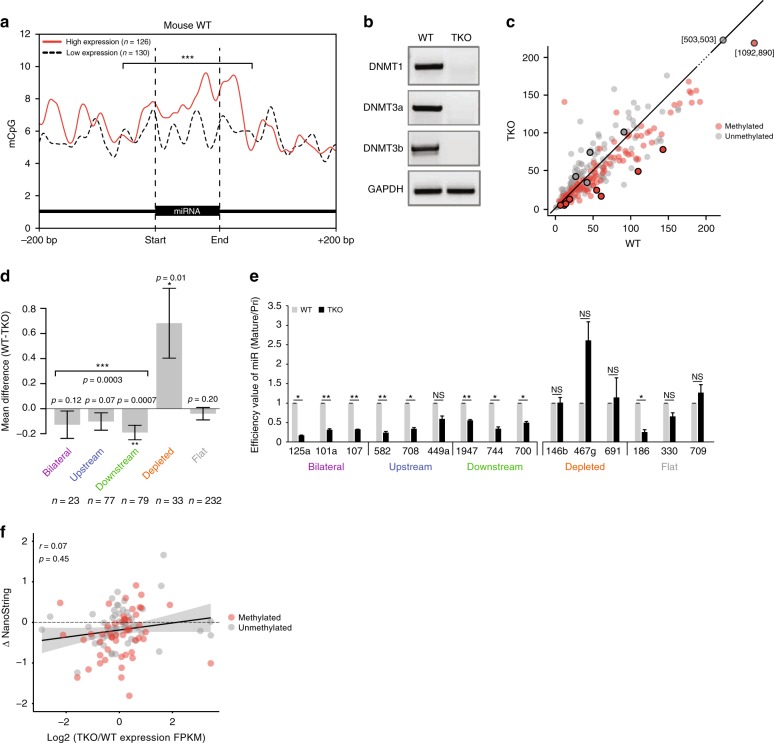
DNA methylation affects miRNA biogenesis. **a** Mean methylated CpG signal plotted across 100 bp of miRNA region and an additional 200 bp of the flanking regions for top and bottom quartiles of miRNA expression. The miRNA region (100 bp) and 25 flanking bp between the two groups were tested for statistical significance. ***p < 0.0001; *t*-test. **b** Quantification of DNMT genomic presence by PCR for WT and TKO mouse ESCs. *GAPDH* was used as the loading control. Source data are provided as a Source Data file. **c** NanoString microarray miRNA expression data in WT vs. TKO cells. Representative miRNAs used for validation are highlighted with open black circles. **d** Plot of the mean differences in mature miRNA expression in WT vs. TKO mouse ESCs. miRNA expression was compared between WT and TKO cells within each group and for all the methylated groups together using one-sided paired *t*-test. *p*-values are given above each bar. **e** Efficiency values, calculated as the level of mature miRNA divided by the level of pri-miRNA for the methylated miRNAs and unmethylated miRNAs in WT and TKO mouse ESCs. Data were normalized to *RPLP0* for pri-miRNAs and U6 for mature miRNAs. Error bars represent ± SEM (*n* = 3). * represents *p* < 0.05; ** represents *p* < 0.01; NS = Not Significant; *t*-test. Source data are provided as a Source Data file. **f** Plot of differential gene expression (log2 FPKM of WT/TKO) vs. differential mature miRNA expression as measured by NanoString analysis. *R*- and *p*-values of Pearson’s product-moment correlation are given.

In order to examine the influence of DNA methylation on biogenesis of miRNAs, we analyzed the expression levels of mature miRNAs in mouse ESCs (see methods for specific GEO accession numbers). We selected miRNAs from the top and bottom quartiles with respect to miRNA expression, and we plotted the frequencies of CpG methylation across their loci (Fig. 2a). Around the miRNA borders, levels of CpG methylation significantly correlated with levels of miRNAs expression, strengthening the notion that methylation is involved in miRNA biogenesis.

We then analyzed miRNA expression in mouse ESCs in which all the three genes encoding DNMTs are knocked out^50^. These triple-knockout (TKO) cells lack any DNA methylation activity (Fig. 2b). We compared the expression of methylated miRNAs and unmethylated miRNAs in wild-type (WT) and TKO cells. Methylated miRNAs were mostly downregulated, whereas unmethylated miRNAs did not exhibit a specific trend (Fig. 2c). Next, we compared the mean expression of each miRNA group between WT and TKO mouse ESCs. In both cell types, we measured the ratio of mature miRNA to pri-miRNA and found that levels of the methylated groups (bilateral, upstream, and downstream) were significantly downregulated (Fig. 2d). Interestingly, the levels of the miRNAs in the depleted group were significantly upregulated in TKO cells, whereas the expression of miRNAs in the flat group did not differ (Fig. 2d). Since the depleted group is one of the smallest, it is much more susceptible to outliers than are the other groups. Moreover, additional factors that influence DNA methylation probably regulate expression of these miRNAs, and expression of these factors themselves might be influenced by levels of DNA methylation. For example, it might be that methylation removal influences Drosha activity or that Dicer or other factors that are active in the cytoplasm might impact expression of mature miRNAs.

We next validated the observations made at the group level by measuring mature miRNA levels of representative miRNAs from each group (Supplementary Data 1): bilateral (miR-125a, miR-101a, and miR-107), upstream (miR-582, miR-708, and miR-449a), downstream (miR-1947, miR-744, and miR-700), depleted (miR-146b, miR-467g, and miR-691), and flat (miR-186, miR-330, and miR-709). The miRNAs that were chosen for experimental validation are highlighted in Fig. 2c. All miRNAs selected for validation are intronic. The choice of intronic miRNAs excludes a possible effect due to differences in promoter methylation. The methylated miRNAs that were chosen displayed differed considerably in expression when levels in WT and TKO cells were compared, whereas unmethylated miRNAs did not. Consistent with the global data, in the TKO cells most miRNAs from the methylated groups were significantly lower than in WT cells, and one methylation depleted miRNA was upregulated (Fig. 2e and Supplementary Fig. 3).

To rule out the possibility that the differences in mature miRNAs levels between WT and TKO cells were due to differences in gene expression, we compared the differences in mature miRNAs levels to differences in transcript levels. We found no correlation between host transcript levels and mature miRNA levels for any of the loci evaluated (Fig. 2f). The downregulation of methylated miRNAs in cells lacking this epigenetic mark implies that methylation is involved in miRNA biogenesis.

### Drosha genomic occupancy is sensitive to DNA methylation

**Fig. 3:**
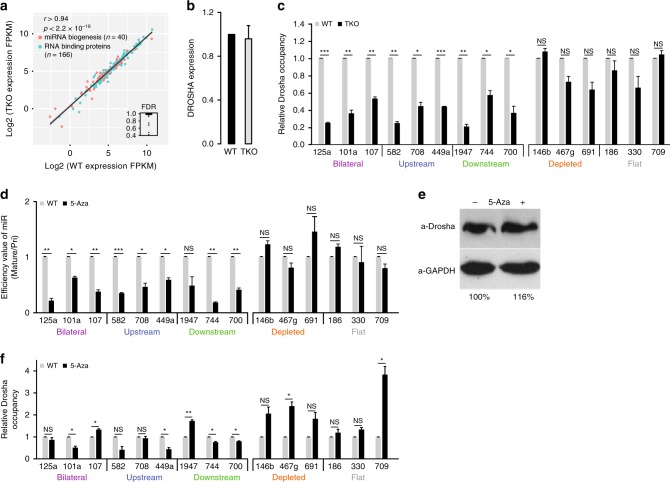
Drosha genomic occupancy is sensitive to DNA methylation. **a** Plot of expression of transcripts encoding miRNA biogenesis proteins (*n* = 40) and other RNA-binding proteins (*n* = 166) in WT vs. TKO cells. *R*- and *p-*values of Pearson’s product-moment correlation are given. **b** *Drosha* mRNA levels in WT and TKO mouse ESCs quantified by qRT-PCR. Bars represent means of three independent experiments. Values were normalized to *tubulin*. **c** Relative Drosha occupancy over miRNA genomic regions in WT and TKO mouse ESCs determined by ChIP with an antibody against Drosha. Immunoprecipitated DNA was quantified by qRT-PCR with primers spanning the indicated pre-miRNA sequences. Data were normalized to input DNA. **d** Efficiency of miRNA biogenesis measured as the ratio of mature miRNA to pri-miRNA expression levels for the indicated miRNAs in WT mouse ESCs upon treatment with 2.5 µM 5-Aza relative to vehicle control. Data were normalized to *RPLP0* for pri-miRNAs and RNU6 for mature miRNAs. **e** Drosha protein levels in untreated control WT mouse ESCs and cells treated with 2.5 µM 5-Aza. GAPDH was used as the loading control. **f** Relative Drosha occupancy over miRNA genomic regions in untreated WT mouse ESCs and cells treated with 2.5 µM 5-Aza determined by ChIP with an antibody against Drosha. Immunoprecipitated DNA was quantified by qRT-PCR with primers spanning the indicated pre-miRNA sequences of the methylated and unmethylated groups. Data were normalized to input DNA. All Error bars represent ± SEM (*n* = 3). * represents *p* < 0.05; ** represents *p* < 0.01; *** represents *p* < 0.001; NS = not significant; *t*-test. Source data are provided as a Source Data file.

To exclude the possibility that the differential expression in TKO cells vs. WT cells results from differential expression of proteins involved in miRNA biogenesis or RNA-binding proteins, we examine expression levels of relevant mRNAs in TKO cells compared to WT cells (see methods for specific GEO accession numbers). Of 180 genes encoding RNA-binding proteins that were identified by Treiber et al.^51^ in humans, we found that 166 have homologs in mice. There were not significant differences in expression of genes encoding proteins involved in miRNA biogenesis or RNA-binding proteins when WT and TKO cells were compared (Fig. 3a), suggesting that DNA methylation directly influences miRNA biogenesis.

To further validate these results, we performed quantitative reverse transcription-PCR (qRT-PCR) to examine *Drosha* expression in WT and TKO cells and found no significant difference (Fig. 3b). Next, we examined Drosha occupancy over miRNA loci by performing chromatin immunoprecipitation (ChIP) assays in WT and TKO cells. The presence of Drosha was significantly lower at regions encoding miRNAs from the methylated groups in the TKO cells compared to WT cells (Fig. 3c). In contrast, there was no difference in Drosha occupancy over loci encoding miRNAs from the depleted or flat groups (Fig. 3c).

To further evaluate the mechanistic link between DNA methylation and miRNA biogenesis we treated WT cells with 5-aza-2’-deoxycytidine (5-Aza), which causes demethylation of genomic DNA^52^ and a general increase in gene expression^53^. In order to specifically determine the effect of 5-Aza treatment on miRNA biogenesis, we measured the levels of mature miRNAs relative to the levels of pri-miRNA with and without 5-Aza treatment (Supplementary Fig. 4). The expression of methylated miRNAs was significantly decreased with treatment, implying that biogenesis was inhibited upon demethylation (Fig. 3d). This was not the case for miRNAs from unmethylated groups (Fig. 3d and Supplementary Fig. 4). To verify that the effect of 5-Aza treatment was not a result of different levels of Drosha expression, we analyzed Drosha protein levels. We found no significant difference in Drosha amounts between 5-Aza-treated and untreated cells (Fig. 3e).

Next, we examined Drosha occupancy over miRNA loci by performing ChIP assays in WT ESCs after treatment with 5-Aza. Upon 5-Aza treatment Drosha occupancy was significantly decreased at most of the regions encoding miRNAs from the methylated groups (Fig. 3f). In contrast, there was either no difference or an increase in Drosha occupancies over miRNAs from the depleted and flat groups (Fig. 3f). This strengthens our hypothesis that DNA methylation directly affects Drosha binding and, thus, miRNA biogenesis efficiency.

### MeCP2 binding to methylated miRNA loci slows Pol II elongation to allow miRNAs biogenesis

To determine the mechanism by which DNA methylation influences Drosha occupancy and miRNA biogenesis, we considered several pieces of evidence. First, our data demonstrate that regions that encode miRNAs from the methylated groups are occupied by nucleosomes at significantly higher frequency (Fig. 1e, f), and it is known that nucleosome occupancy^54^ and DNA methylation^55^ influence the rate of Pol II elongation. Second, Pol II elongation rate modulates the inclusion of alternatively spliced exons^41,42^. Finally, Drosha is associated with Pol II^56^. This led us to examine the involvement of Pol II in the processing of the methylated and unmethylated miRNAs. We hypothesized that DNA methylation at miRNA boundaries results in Pol II pausing, which provides time for the miRNA-processing machinery to access the newly synthesized pri-miRNA, in the same way that Pol II pausing enhances exon selection by the splicing machinery^41,42^.

**Fig. 4:**
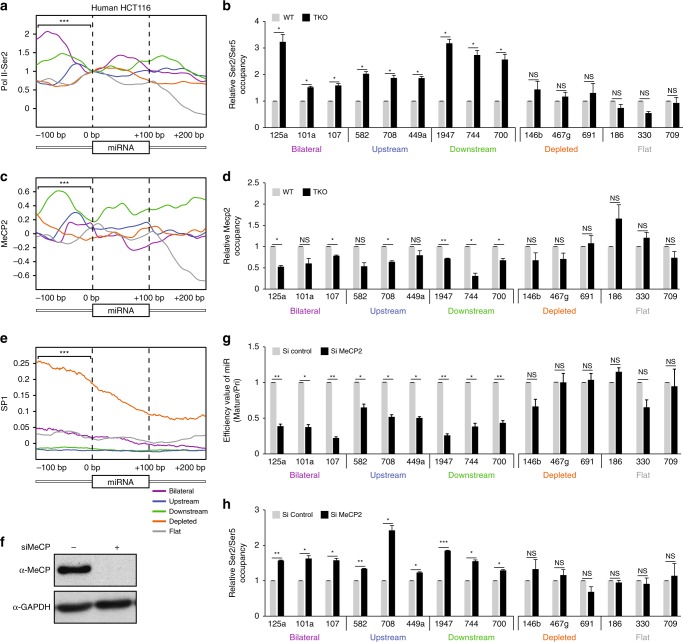
MeCP2 binding at methylated miRNA loci slows Pol II allowing miRNAs biogenesis. **a** Pol II-pSer2 occupancies for each group of miRNAs across 100 bp of miRNA region and 100 bp of the flanking regions. Regions upstream of the 5‘ ends of the pre-miRNAs between all methylated groups vs. the unmethylated groups were tested. All tests were statistically significant. ***p < 0.001; *t*-test. **b** Relative Pol II-pSer2 to Pol II-pSer5 (Ser2/Ser5) occupancies analyzed by ChIP over select miRNA regions in WT and TKO mouse ESCs. Immunoprecipitated DNA was quantified by qRT-PCR using primers spanning the indicated pre-miRNA sequences. Data were normalized to input DNA. **c** MeCP2 oc**c**upancies in regions upstream of start sites between all methylated groups vs. the two unmethylated groups were tested. All tests were statistically significant except for the test between the bilateral and the depleted groups. ***p < 0.001; *t*-test. **d** Relative MeCP2 occupancy over miRNA genomic regions in WT and TKO ESCs determined by ChIP analysis. Immunoprecipitated DNA was quantified by qRT-PCR using primers spanning the indicated pre-miRNA sequences. Data were normalized to input DNA. **e** SP1 occupancy for each group of miRNAs. The regions upstream of the start sites between all methylated groups vs. the unmethylated groups were tested. All tests were statistically significant. ***p < 0.001; *t*-test. **f** MeCP2 protein levels in WT mouse ESCs upon treatment with siMeCP2 or siControl. GAPDH was used as a loading control. **g** Efficiency values for the methylated miRNAs and unmethylated miRNAs from WT mouse ESCs upon treatment with siMeCP2 or siControl. Data were normalized to *RPLP0* for pri-miRNAs and U6 for mature miRNAs. **h** Relative Pol II-pSer2 to Pol II-pSer5 (Ser2/Ser5) occupancy analyzed by ChIP in WT mouse ESCs upon treatment with siMeCP2 or siControl. Immunoprecipitated DNA was quantified by qRT-PCR. Data were normalized to input DNA. All error bars represent ± SEM (*n* = 3); * represents *p* < 0.05; ** represents *p* < 0.01; *** represents *p* < 0.001; NS = not significant; *t*-test. Source data are provided as a Source Data file.

To test this theory, we analyzed ChIP-seq data of total Pol II and Pol II-pSer2 derived from human HCT116 cell extracts^29,57^. To account for differences in gene expression, we normalized each signal to the value that was measured at the first base of each miRNA. Total Pol II and Pol II-pSer2 occupancies at the boundaries of the human miRNAs were higher for the methylated groups compared to the unmethylated groups of miRNAs (Supplementary Fig. 5a and Fig. 4a, respectively). Additionally, we analyzed previously published ChIP-seq data of total Pol II ChIP-seq in mouse ESCs^58^. Consistent with the results in human cells, we observed a higher Pol II signal at the boundaries of the miRNAs from the methylated groups compared to those from the unmethylated groups (Supplementary Fig. 5b).

Pol II phosphorylation at Ser2 is associated with elongation, whereas phosphorylation at Ser5 is associated with pausing^45^, and the ratio between Pol II-pSer2 to Pol II-pSer5 can be used to infer elongation rate^44^. We therefore performed ChIP using antibodies to Pol II-pSer5 and Pol II-pSer2 in mouse WT and TKO cells. A significantly higher elongation rate was observed over loci encoding miRNAs from the methylated groups in TKO cells than in WT cells (Fig. 4b), whereas there was no significant difference for miRNA loci in the depleted or flat groups. This result supports our hypothesis that Pol II pausing at the methylated pre-miRNA boundaries promotes their biogenesis. Taken together, our data suggest that DNA methylation at the borders of pre-miRNAs slows the Pol II elongation rate and thus allows Drosha to access the pre-miRNA-processing sites.

MeCP2 is known to bind 5’-methylcytosine across the genome^22,23^, and this interaction influences Pol II elongation^25,31,59^. It was recently reported that MeCP2 interacts with the Drosha co-factor DGCR8, suggesting that MeCP2 might be involved in miRNA processing^27^. Analysis of MeCP2 ChIP-seq data from human HCT116 cells^59^ revealed significant accumulation near the start sites for miRNAs from the methylated groups but not the unmethylated groups (Fig. 4c), and the signal resembled that of Pol II (Fig. 4a). For the bilateral group we observed only a weak signal, however, this is probably due the low depth of the sequencing.

To test whether MeCP2 is associated with the biogenesis of certain miRNAs, MeCP2 occupancy over miRNAs was examined via ChIP analysis in mouse WT and TKO cells. There was significantly lower MeCP2 occupancy over miRNAs from the methylated groups in TKO compared to WT cells, but there was no significant difference over miRNAs from the unmethylated groups (Fig. 4d). This suggests that MeCP2 is sensitive to DNA methylation at the borders of pre-miRNAs and when methylation is abolished, MeCP2 binding to these regions decreases.

We also analyzed SP1 occupancy over miRNA loci as SP1 is central to maintenance of unmethylated CpG islands^60^, and its occupancy has been shown to be anti-correlated with MeCP2 occupancy^61,62^. By analysis of SP1 ChIP-seq data from human HCT116 cells^59^, we found that SP1 occupancy was significantly higher over miRNAs from the unmethylated groups compared to those from the methylated groups (Fig. 4e), suggesting that SP1 ensures that certain miRNA loci remain unmethylated.

To further investigate the role of MeCP2 in miRNA biogenesis, we inhibited expression of MeCP2 in mouse ESCs cells using an small-interfering RNA (siRNA) complementary to *MeCP2* (siMeCP2, Fig. 4f). MeCP2 deficiency led to significant decreases in levels of mature miRNAs in the methylated groups without altering levels of miRNAs in the unmethylated groups (Fig. 4g and Supplementary Fig. 5c, d). In addition, the Pol II elongation rate over the loci encoding the miRNAs in the methylated groups was significantly higher in cells deficient in MeCP2 compared to control cells, whereas the rate over the loci encoding miRNAs from the unmethylated groups did not differ (Fig. 4h). As a control, we showed that in MeCP2-deficient cells there was a decrease in MeCP2 occupancy over all tested pre-miRNA coding regions (Supplementary Fig. 5e).

**Fig. 5:**
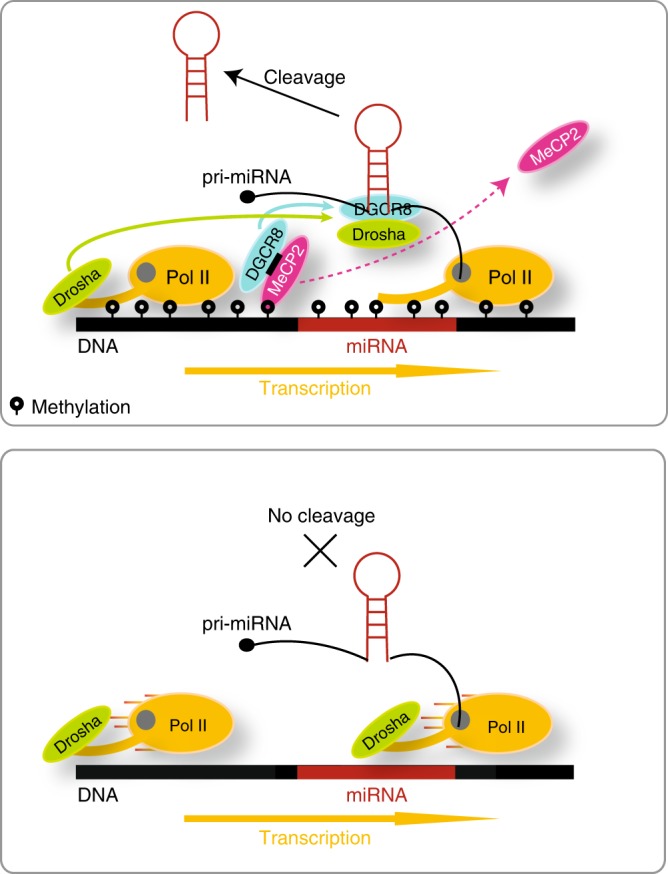
A schematic model of DNA methylation-dependent miRNA processing. Top: MeCP2 binds DGCR8 and slows Pol II elongation. This provides an opportunity for Drosha and DGCR8 to interact with the nascent pri-miRNA. Bottom: In the absence of DNA methylation Pol II-mediated elongation is rapid, and Drosha is unable to bind to the nascent pri-miRNAs.

Since MeCP2 binds over many genomic locations, including non-methylated miRNAs, we expected that depletion of this factor would result in a global decrease in its occupancy genome-wide. However, upon inhibition of MeCP2 expression, MeCP2 occupancy was decreased to a significantly greater extent over methylated miRNA loci than over the non-methylated miRNA loci (Supplementary Fig. 5e). In order to further strength our results, we analyzed miRNA profiling data generated from cerebellum of mice with Rett syndrome (RTT) and normal mouse cerebellum^63^. RTT is a progressive neurodevelopmental disorder caused by mutations in MeCP2^63^. The levels of methylated miRNAs were decreased in cerebellum lacking functional MeCP2, but levels of non-methylated miRNAs were unchanged (Supplementary Fig. 6). Overall, our findings suggest that MeCP2 binds over pri-miRNA coding regions that are methylated at the boundaries, which slows Pol II elongation allowing miRNA biogenesis (Fig. 5).

Finally, to determine whether miRNAs grouped based on methylation status share physiological characteristics, we subjected the groups of miRNAs to KEGG pathway analysis in order to predict biological functions^64^. This analysis took into consideration the biological processes linked to predicted mRNA targets of each miRNA. Interestingly, the miRNAs from loci methylated bilaterally or only upstream or downstream of the miRNA region were more significantly enriched, on average, in cancerous phenotypes than were miRNAs depleted of methylation or those methylated to similar extents across the entire loci (Supplementary Data 2). This suggests that DNA methylation ensures tight regulation of the expression of these critical miRNAs.

## Discussion

This study demonstrates how the activity of the microRNA microprocessor machinery depends on DNA methylation. We discovered that MeCP2 binding to the DNA of methylated miRNAs halts the elongation of the RNA polymerase II, thus enhancing the primary miRNA processing by Drosha and increasing mature miRNA expression. Our data do not eliminate the possibility that additional factors regulating or affected by DNA methylation influence expression of miRNAs. For example, it might be that methylation directly influences Drosha activity. Various factors affecting Drosha cleavage sites on the pri-miRNA, determine Drosha preference for the target^65^; these factors might be influenced by DNA methylation. Moreover, expression of Dicer or other factors that are active in the cytoplasm can be affected by DNA methylation. It is also possible that the secondary or tertiary structures of DNA and RNA might be affected by DNA methylation, and these structures might influence miRNA processing. It was previously shown that secondary structures of transcripts of clustered miRNAs directly dictate which of the miRNAs are processed by Drosha^66^. Secondary structures of mRNAs have also been shown influence translation, and differences can result in atypical phenotypes^67–69^. Other factors like metabolism, diet, lifestyle, and environmental and chemical stressors affect the DNA methylation patterns in mammalian cells^70^ dictating the downstream effect on miRNA expression via Drosha or Dicer^71^, either directly or indirectly.

miRNAs regulate differentiation, proliferation, and apoptosis—processes vital to normal cell development, as well as cancer initiation and progression^72^. Depending on their target genes, miRNAs may function as oncogenes or tumor suppressors^72^. Thus, they can serve as cancer biomarkers^73^ and may have a therapeutic potential^74^. That miRNAs participate in multiple essential processes requires that their expression by tightly regulated. Our data support the hypothesis that methylation of miRNA loci is a pivotal regulator of mature miRNA levels: A high frequency of methylation of regions flanking the pre-miRNA coding sequence results in ameliorated miRNA biogenesis. Interestingly, evaluation of highly methylated miRNAs demonstrated an enrichment in cancer-related phenotypes compared to miRNAs processed from unmethylated loci. For example, miR-182 and miR-135b loci, which are highly methylated upstream of the pre-miRNA coding regions, are present at significantly higher levels breast cancer tumors than normal tissue^75^. Aberrant expression and an oncogenic role of miR-182 have also been demonstrated in gastric^76^, bladder^76^, and prostate cancer^76^. Additional research will be needed in order for fully explore the various regulators of miRNA expression.

## Methods

### Genomic coordinates of miRNAs

Genomic coordinates of human (GRCh37/hg19) and mouse (GRCm38/mm10) miRNAs were downloaded from miRBase^77^ (miRBase v19, http://www.mirbase.org/).

### Cell culture and chemicals

WT R1 mouse ESCs and DNMT TKO ESCs were a kind gift from Professor Eran Meshorer (The Hebrew University of Jerusalem, Israel). TKO ESCs were originally generated in the lab of Professor Masaki Okano (Riken, Japan). WT and TKO R1 cells were cultured in Dulbecco’s Modified Eagle’s Medium (DMEM) supplemented with 15% fetal calf serum, 0.29 mg ml^−1^ stable l-glutamine, 100 units ml^−1^ penicillin, 0.1 mg ml^−1^ streptomycin, 0.1 mM non-essential amino acids, 1 mM sodium pyruvate (all from Biological Industries), 100 μM beta-mercaptoethanol (Sigma Aldrich), and 1000 U ml^−1^ leukemia inhibitory factor (Sigma Aldrich). Cells were maintained on plates coated with 0.2% gelatin (Sigma Aldrich). Cells were incubated at 37 °C in a humidified atmosphere with 5% CO_2_. 5-Aza (Sigma Aldrich) was dissolved according to the manufacturer’s instructions, and cells were treated with 2.5 µM 5-Aza for 24 h. Culture medium was replaced before treatments.

### RNA oligonucleotide transfection

siRNAs designed to hybridize to mouse *Mecp2* and the siControl from the TriFECTa RNAi Kit (Integrated DNA Technologies) were transfected into the WT R1 cells using HiPerFect (QIAGEN) according to the manufacturer’s protocols. Cells were transfected twice with 25 nmol siRNA per culture plate at an interval of 24 h. siRNA sequences are listed in Supplementary Data 3.

### RNA purification and quantitative RT-PCR

Total RNA was extracted using Trizol (Ambion) according to manufacturer’s instructions. RNA quality was assessed by measuring absorbance at 260 nm relative to 280 nm. For qRT-PCR analysis of mature miRNAs, 10 ng of total RNA was used in a TaqMan miRNA assay according to the manufacturer’s protocols (Applied Biosystems). Expression levels were normalized to RNU6 snRNA expression. All reactions were performed in duplicate. For pri-miRNA analyses, 50 ng of total RNA was subjected to one-step RT-PCR with MultiScribe Reverse Transcriptase (Invitrogen) and FastStart Universal SYBR Green FastMix (Quantabio). Relative expression was normalized to *RPLP0*. All reactions were performed in duplicate. Error bars represent mean values ± SEM. All primer sequences are listed in Supplementary Data 3.

### Chromatin immunoprecipitation

Approximately 3 × 10^7^ cells (R1 or TKO) were crosslinked for 10 min in 1% formaldehyde. The crosslinker was quenched by incubation with a final concentration of 125 mM glycine for 5 min at 37 °C. Cells were washed twice with ice-cold PBS gently and scrapped in 4 ml chilled PBS using a cell scrapper. Cells were centrifuged at 2700 rpm for 2 min at 4 °C. The pellet was re-suspended in 4 ml ice-cold homogenization buffer (10 mM Tris-HCl pH 7.4, 15 mM NaCl, 60 mM KCl, 1 mM EDTA, 0.1% SDS, 5% sucrose, and complete protease inhibitor cocktail (Roche)) and transferred to a pre-chilled Dounce homogenizer kept on ice. The cells were homogenized by applying six strokes on the homogenizer, and the samples were transferred to fresh 15-ml tubes. To the bottom of the tube was added a 2-ml sucrose pad (10 mM Tris-HCl, pH 7.4, 15 mM NaCl, 60 mM KCl, 5% sucrose, and complete protease inhibitor cocktail (Roche)). The tubes were centrifuged at 2000 rpm for 5 min at 4 °C and the supernatant was discarded. Pellets containing nuclei were re-suspended in 600 μl Lysis Buffer (1% SDS, 10 mM EDTA, 50 mM Tris-HCl, pH 8.1, complete protease inhibitor cocktail (Roche)) and sonicated with a Vibra-Cell VCX600 (Sonics & Materials) for 40 min (1.5-second pulses at 40% amplitude, 9.9-second rest) to obtain an average DNA length of 150–350 bp. After centrifugation at 20,000 × *g* for 8 min at 4 °C, the supernatant was diluted with 4.5 ml (1:10) Dilution Buffer (0.01% SDS, 1.1% Triton X-100, 1.2 mM EDTA, 16.7 mM Tris-HCl, pH 8.1, 167 mM NaCl, complete protease inhibitor cocktail (Roche)) and aliquoted for immunoprecipitation (IP); 100 µg chromatin from cells was used per IP reaction. Input material (100 μl) was also aliquoted for later analysis. Prior to use, protein A/G Plus-agarose beads (Santa Cruz Biotechnology) were washed twice with RIPA Buffer (0.1% deoxycholate, 0.1% SDS, 1% Triton X-100, 10 mM Tris-HCl, pH 8.1, 1 mM EDTA, 140 mM NaCl). For each IP sample, 50 µl of protein A/G Plus-agarose beads were re-suspended in 350 μl Blocking Buffer (PBS, 0.5% TWEEN, 0.5% BSA) and incubated for 2 h at 4 °C with the antibody of interest. The conjugated beads were added to each IP tube, and samples were incubated with rotation for 16 h at 4 °C. The antibodies used were normal rabbit IgG (3 μg antibody per 100 μg chromatin; sc-2027, Santa Cruz Biotechnology), anti-Pol II pSer2 (3 μg antibody per 100 μg chromatin; ab5095, Abcam), anti-Drosha (3 μg antibody per 100 μg chromatin; ab12286, Abcam), anti-RNA polymerase II CTD repeat YSPTSPS pSer5 (3 μg antibody per 100 μg chromatin; ab5408, Abcam), anti-RNA polymerase II CTD repeat YSPTSPS pSer2 (3 μg antibody per 100 μg chromatin; ab5095, Abcam), and anti-MeCP2 (8 μg antibody per 100 μg chromatin; #3456, Cell Signaling). The beads were washed five times with RIPA buffer (0.1% deoxycholate, 0.1% SDS, 1% Triton X-100, 10 mM Tris-HCl, pH 8.1, 1 mM EDTA, 140 mM NaCl), twice with RIPA-high-salt buffer (0.1% deoxycholate, 0.1% SDS, 1% Triton X-100, 10 mM Tris-HCl, pH 8.1, 1 mM EDTA, 360 mM NaCl), twice with LiCl wash buffer (250 mM LiCl, 0.5% NP-40 (Sigma Aldrich), 0.5% deoxycholate, 1 mM EDTA, 10 mM Tris-HCl, pH 8.1), and twice with TE buffer (10 mM Tris-HCl, pH 8.1, 1 mM EDTA). DNA was eluted from the beads with 200 μl Elution buffer (0.5% SDS, 300 mM NaCl, 5 mM EDTA, 10 mM Tris-HCl, pH 8.1) using a 30-min incubation in a thermo-shaker at 65 °C. Elution buffer (100 μl) was added to input sample. From this stage on, input tubes were processed similarly to IP tubes: 1 µl of 10 mg ml^−1^ RNase A (Sigma Aldrich) was added to the supernatant, samples were incubated for 30 min at 37 °C, 1.5 µl Proteinase K (New England Biolabs) was added, and samples were incubated for 16 h at 65 °C. DNA was purified using phenol:chloroform:isoamyl alcohol (25:24:1; Sigma Aldrich) extraction according to the manufacturer’s instructions. ChIP samples were deep sequenced on a HiSeq 2500 in 50-bp single-end reads at the Technion Genome Center in Israel Institute of Technology (Haifa, Israel). qRT-PCR was then performed on ChIP samples to amplify specific pri-miRNA regions. Relative expression was normalized to respective input samples. All reactions were performed in duplicate. Error bars represent mean values ± SEM. All primer sequences are listed in Supplementary Data 3.

### Western blotting

Proteins were extracted into a hypotonic lysis buffer (50 mM Tris-HCl, pH 7.5, 1% NP-40, 150 mM NaCl, 0.1% SDS, 0.5% deoxycholic acid, 1 mM EDTA) containing protease inhibitor (Roche) and phosphatase inhibitor cocktails I and II (Sigma Aldrich). Proteins were separated in 10% SDS-PAGE and then electroblotted onto a Protran nitrocellulose transfer membrane (Whatman). The following antibodies were used: anti-Drosha (1:200; sc-31159, Santa Cruz Biotechnology), anti-MeCP2 (1:1000; #3456, Cell Signaling) and anti-GAPDH (1:1000; A00191-40, GenScript,). Blots were incubated for 12 h at 4 °C, followed by incubation with appropriate HRP-conjugated secondary antibodies: donkey anti-goat (1:30,000; ab97120, Abcam) or donkey anti-rabbit (1:30,000; ab97064, Abcam).

### DNA methylation and data analysis

Methylation profiles for mouse and human were taken from the ENCODE project. We used whole-genome bisulfite sequencing (WGBS) data from postnatal 0 day mouse intestine (ENCSR353IFP), heart (ENCSR397YEG), kidney (ENCSR128HOP), liver (ENCSR550CYA), and lung (ENCSR409HKJ). Raw WGBS reads from mouse ESCs were downloaded from the GEO (GSE82125) and were processed using Bismark^78^. For human methylation profiles, we used WGBS data from ENCODE for brain (ENCSR145HNT), heart (ENCSR699ETV), liver (ENCSR351IPU), lung (ENCSR797TEV), skin (ENCSR128RMY), intestine (ENCSR522UKJ), and ESCs (ENCSR617FKV). The DNA methylation patterns of regions flanking the pre-miRNA coding regions were used to define the methylated groups and the unmethylated groups. The methylated miRNAs were identified as miRNAs with high-mean methylation levels 25 bp upstream of the pre-miRNA start positions and 25 bp downstream of the pre-miRNA end positions. The depleted miRNAs were defined as having no methylation within the pre-miRNA regions or in the regions 250 bp upstream or downstream of the pre-miRNA coding regions. The rest of the miRNAs constitute the flat group. The same algorithm was used to identify methylated and unmethylated miRNAs in mouse and in human cell lines.

### Additional datasets

Conservation scores for human and mouse miRNAs were downloaded from UCSC mm10.60way.phastCons for mouse and hg19.100way.phastCons for human. The MNase-seq data from mouse R1 and human ESCs were taken from the GEO (GSE64910 and GSE76083, respectively). ChIP-seq datasets for human HCT116 cells were taken from the GEO (MeCP2, GSM1154509; Pol II pSer2, GSE47677; and SP1, GSM1010902). ChIP-seq of total Pol II in mouse WT cells was downloaded from the GEO (GSM1446977). We downloaded raw reads and mapped them to the mouse (GRCm38/mm10) and human (GRCh37/hg19) reference genomes using bowtie 2^79^. Reads with mapping quality < 30 were discarded using Samtools (http://www.htslib.org/). We used bam2wig.pl (http://search.cpan.org/~tjparnell/Bio-ToolBox-1.44/) to normalize base coverage to the total number of mapped reads and to construct a standard UCSC BigWig file. We used bwtool^80^ to extract values from BigWig files into BED files with coordinates. For ChIP-seq we subtracted the input signal from the ChIP-seq signal, and for MNase-seq we extended each read to the length of 147 bp. BigWig files for GC and CpG content for both human and mouse were created using in-house Perl scripts.

### Gene and miRNA expression

Gene expression data for WT R1 mouse ESCs and DNMT TKO R1 mouse cell lines were taken from NCBI/GEO record GSE6491021. RNA-seq reads were aligned to the mouse reference genome (GRCm38/mm10) using TopHat^81^ with default parameters. Expression of genes in fragments per kilobase of transcript per million mapped reads (FPKM) was calculated via cufflinks^82^ with default parameters. miRNAs expression in mouse WT R1 ESCs and TKO R1 cells was evaluated in house using a NanoString microarray (full microarray data at the GEO server: GSE124879). Normalization of the data was carried out using the NanoString nSolver Analysis software. miRNA profiling data from cerebellum of mice with RTT and normal cerebellum were taken from the GEO (GSE24320).

### miRNA biogenesis and RNA-binding proteins

The genes encoding RNA-binding proteins were selected based on the Treiber et al. study^51^. Of 180 genes encoding RNA-binding proteins that were identified by Treiber et al. in humans, we found 166 that also exist in mice. microRNA biogenesis genes were taken from GeneCards^83^ (https://www.genecards.org/).

### miRNA physiological function

Target genes of murine miRNAs were found using Tarbase, a large manually curated target database, and analyzed for enriched KEGG pathways using mirPath software (http://snf-515788.vm.okeanos.grnet.gr/) taken from DIANA TOOLS (http://diana.imis.athena-innovation.gr/DianaTools/index.php?r = site/index). The *p*-values of cancer-related pathways were summed for both groups.

### Statistics

Statistical analysis was done using R (version 3.2.1).

### Reporting summary

Further information on research design is available in the Nature Research Reporting Summary linked to this article.

## Supplementary information

Supplementary Information

Reporting Summary

Description of Additional Supplementary Files

Supplementary Data 1

Supplementary Data 2

Supplementary Data 3

## Data Availability

A reporting summary for this Article is available as a Supplementary Information file. The source data underlying Figs. 2b, e, 3b, c, d, e, f, 4b, d, f, g, and 4 h and Supplementary Figs. 3a, 3b, 4a, 4b, 5c, 5d, and 5e are provided as a “Source Data file”. miRNA microarray data in support of this study have been deposited at the GEO with primary accession number GSE124879. All other data supporting the findings of this study are available from the corresponding author on request.

## Source data

Source Data

## Code availability

All codes are available upon reasonable request.
