# Supplementary material for: DNA methylation directs microRNA biogenesis in mammalian cells
Source: Nat Commun. 2019 Dec 11;10:5657. doi: 10.1038/s41467-019-13527-1 (PMC6906426; doi:10.1038/s41467-019-13527-1)
Supplement: Supplementary file 1 — Supplementary Information [file 41467_2019_13527_MOESM1_ESM.pdf]

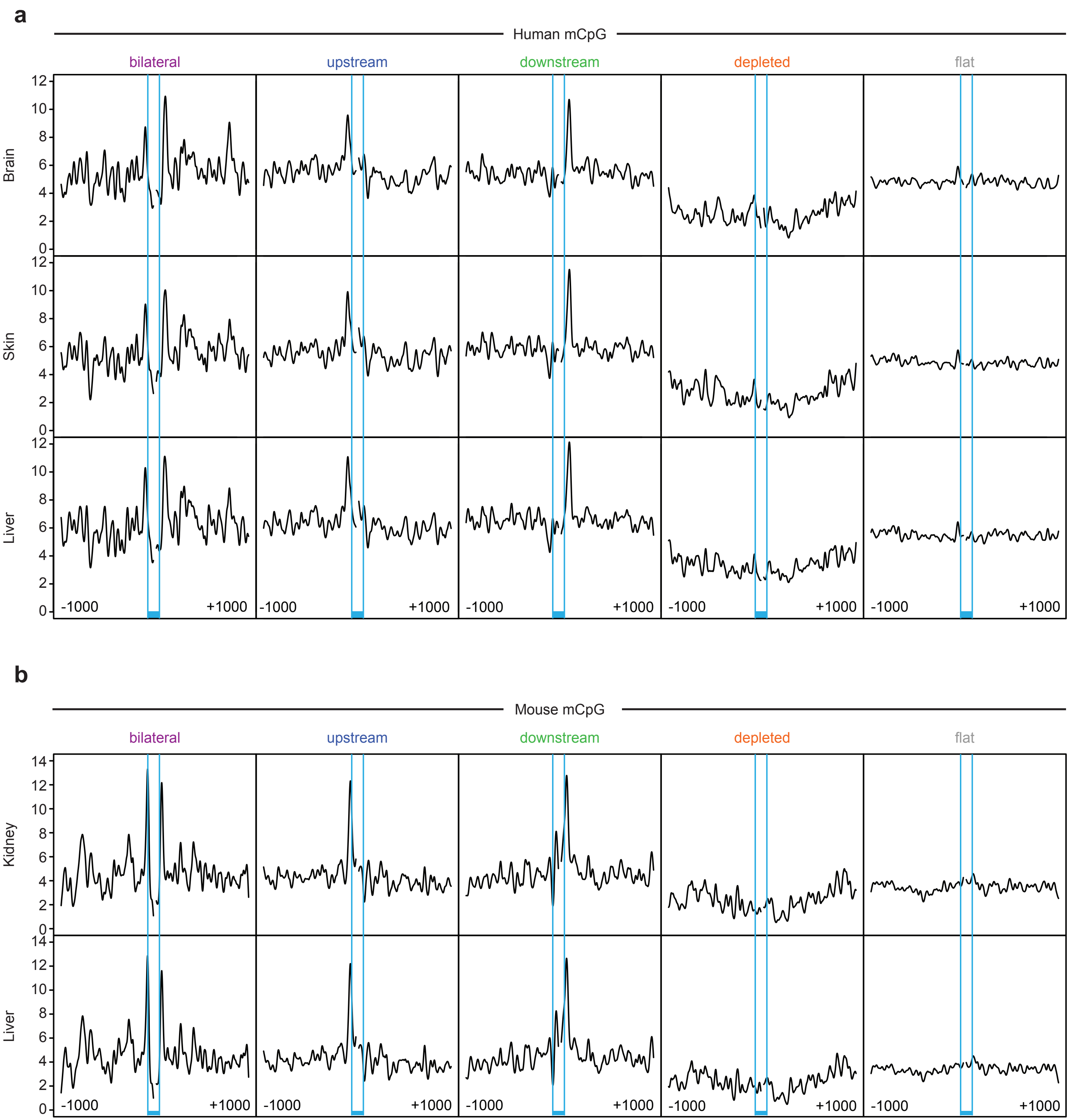

Supplementary Figure 1

Supplementary Fig. 1. Methylation marks the location of pre-miRNA boundaries. **a-b** miRNA methylation profiles as in Fig. 1 for a) human brain, skin, and hepatocytes and b) mouse kidney and liver.

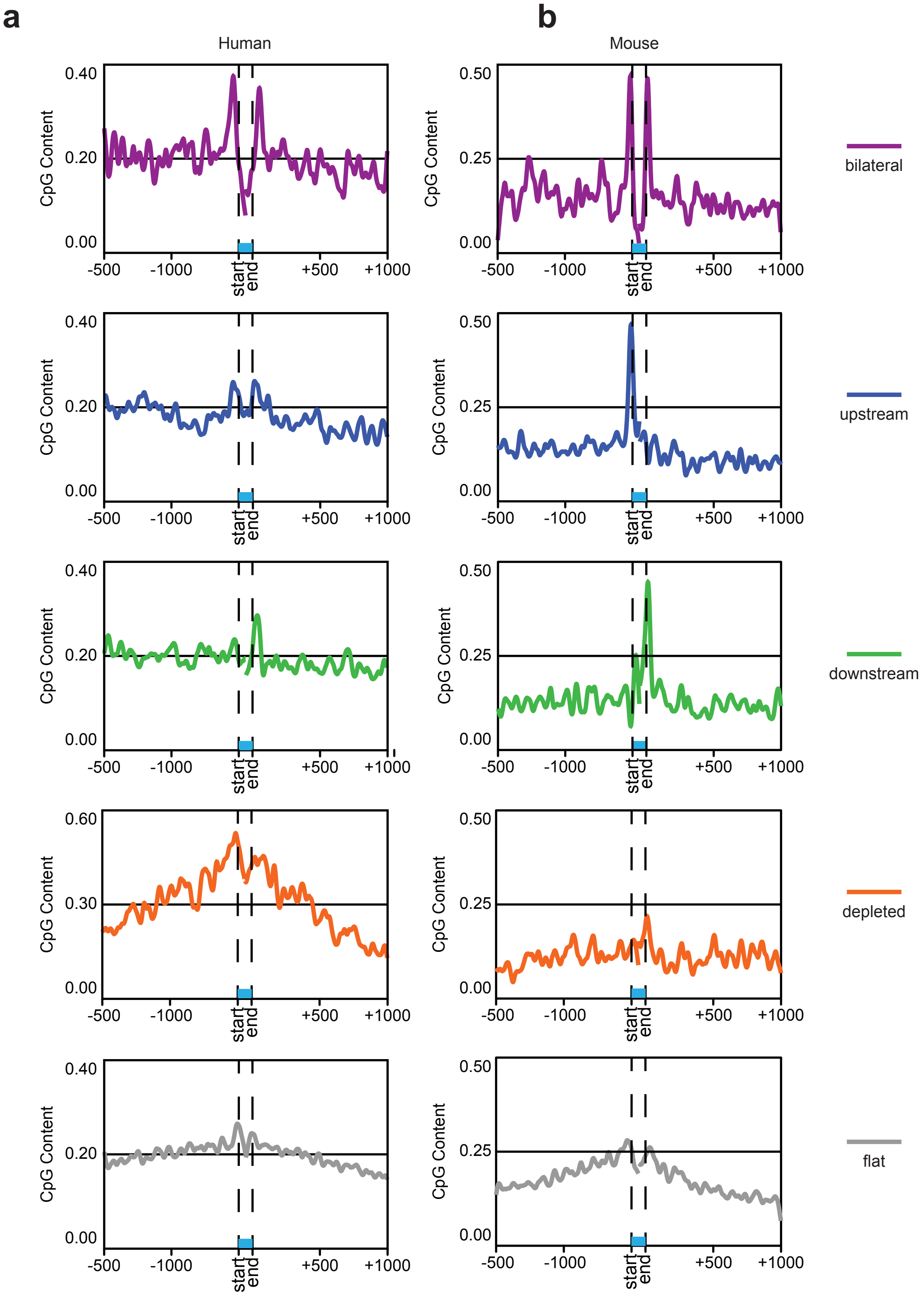

Supplementary Figure 2

Supplementary Fig. 2. DNA methylation affects miRNA biogenesis. **a-b** Mean CpG content for each of the miRNA groups in a) human and b) mouse across 100 bp of the miRNA coding region and 1,000 bp upstream and downstream. Light blue lines indicate miRNA borders.

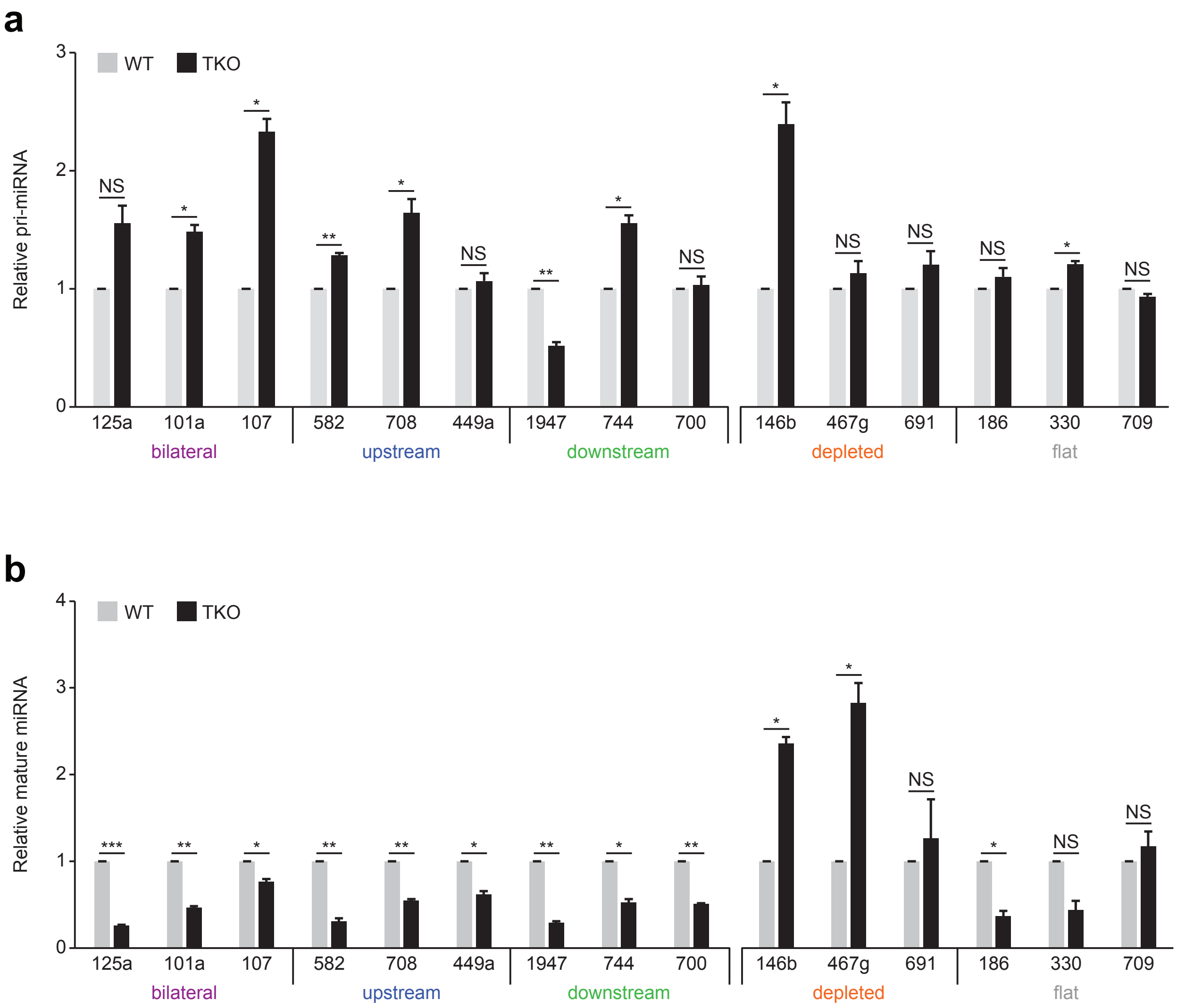

Supplementary Figure 3

Supplementary Fig. 3. DNA methylation affects miRNA biogenesis. **a** Relative pri-miRNA expression levels of methylated and unmethylated miRNAs from WT and TKO mouse ESCs. Data were normalized to *RPLP0*. **b** Relative mature miRNA expression levels for the methylated and unmethylated groups in WT and TKO mouse ESCs. Data were normalized to U6. All error bars represent ± SEM (n=3). * represents P < 0.05; ** represents p < 0.01; ***p represents < 0.001; NS = Not Significant; t-test. Source data are provided as a Source Data file.

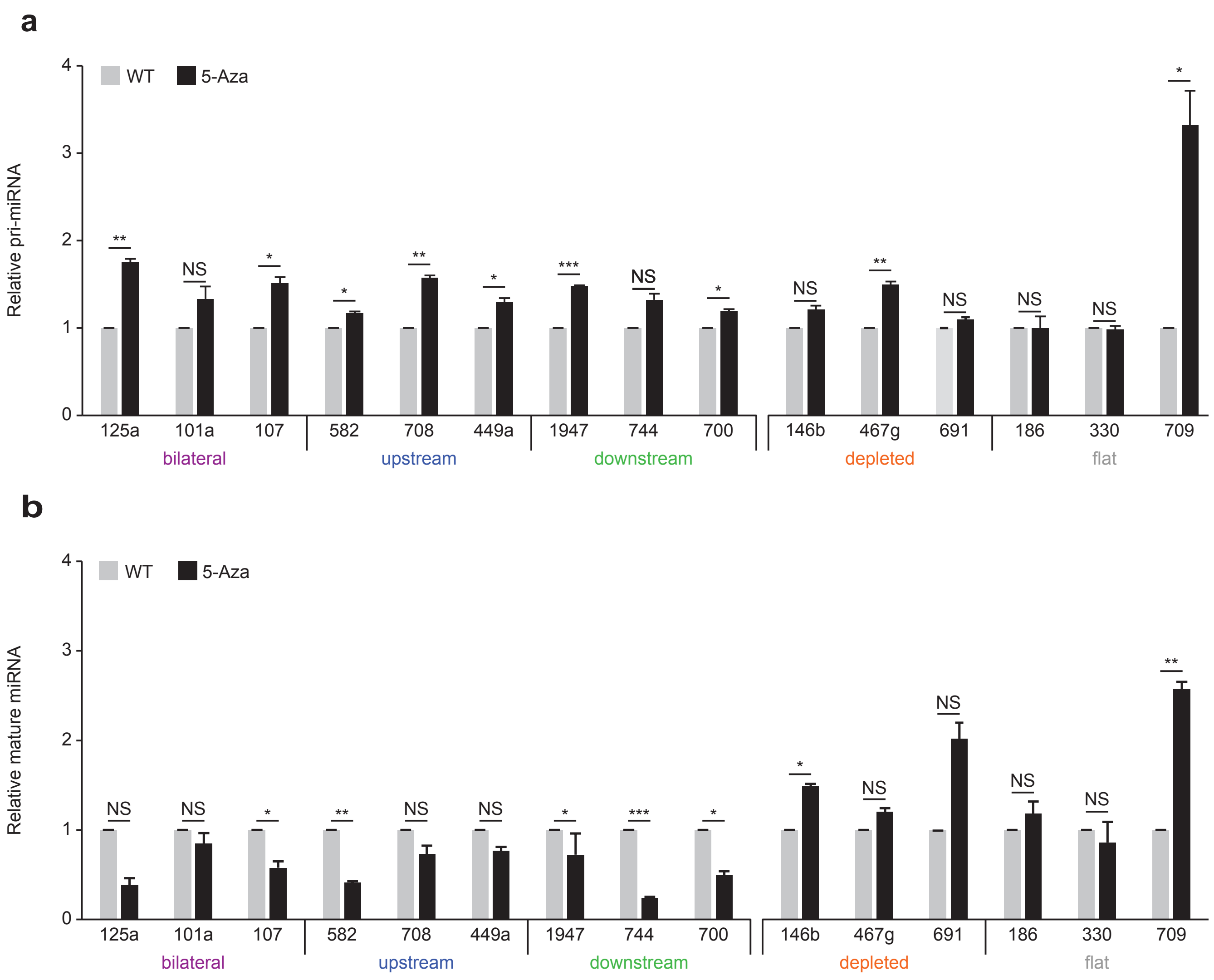

Supplementary Figure 4

Supplementary Fig. 4. DNA methylation affects miRNA biogenesis. **a** Relative pri-miRNA expression levels for the miRNA groups in untreated WT mouse ESCs and cells treated with 2.5 µM 5-Aza. Data were normalized to *RPLP0*. **b** Relative mature miRNA expression levels for the miRNA groups in untreated WT mouse ESCs and cells treated with 2.5 µM 5-Aza. Data were normalized to RNU6. All error bars represent ± SEM (n=3); * represents $p < 0.05$; ** represents $p < 0.01$; ***p represents < 0.001; NS = Not Significant; t-test. Source data are provided as a Source Data file.

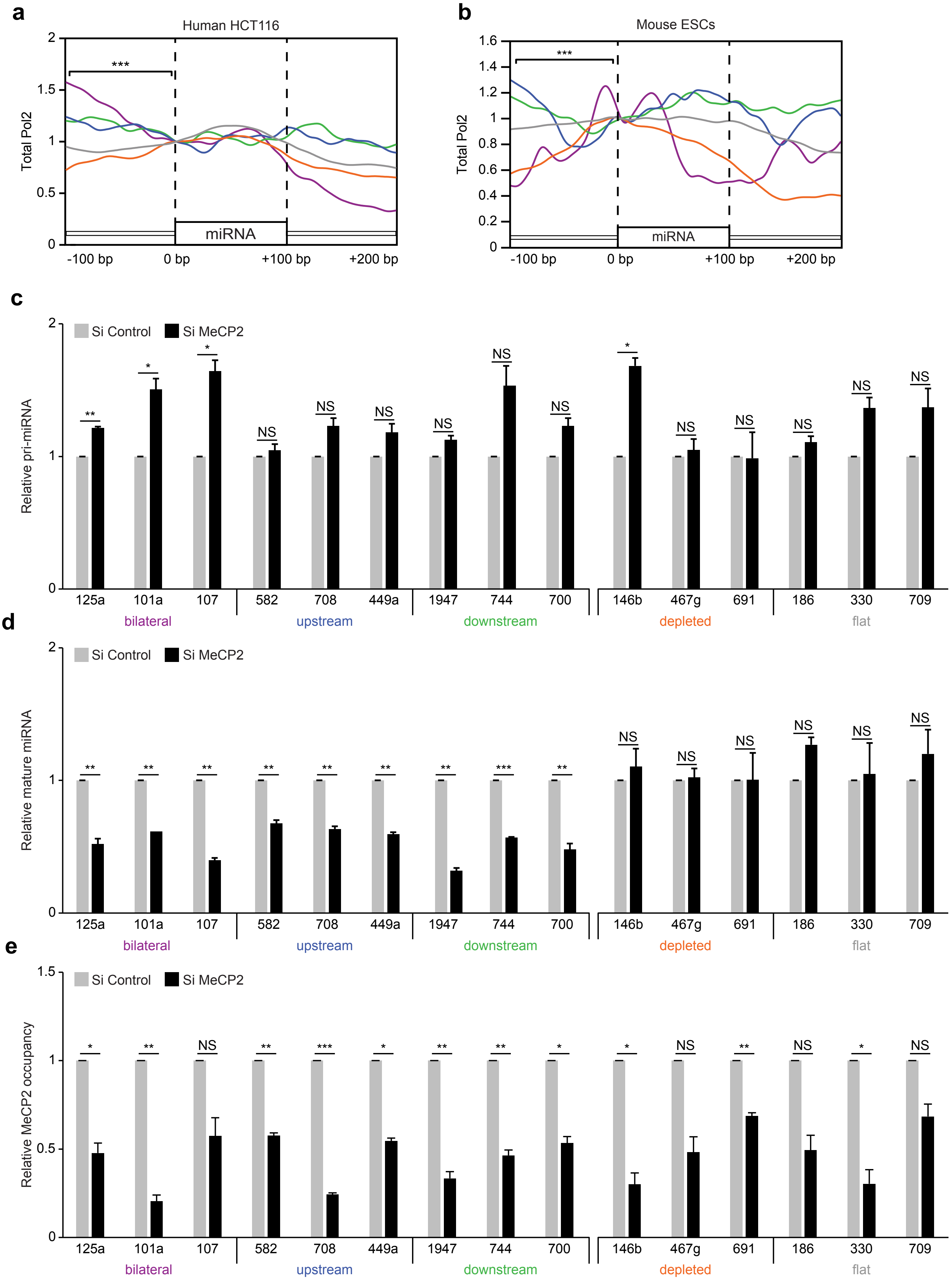

Supplementary Figure 5

Supplementary Fig. 5. DNA methylation reduces Pol II elongation rate. **a** Total Pol II occupancy in human HCT116 cells for each group of miRNAs across 100 bp of miRNA region and 100 bp of the flanking regions. The regions upstream of the start sites of methylated groups of miRNAs (bilateral, upstream, and downstream) were compared to those of the depleted and flat groups. **b** Total Pol II occupancy in mouse WT cells for each group of miRNAs across 100 bp of miRNA region and 100 bp of the flanking regions. The regions upstream of the 5' ends of pre-mRNAs for all methylated groups vs. the unmethylated groups were tested. All tests were statistically significant except for the test between the bilateral and the depleted groups and the test between the upstream and the flat groups. **c** Relative MeCP2 occupancy over miRNA genomic regions in WT mouse ESCs upon treatment with siMeCP2 or siControl. Treated cells were subjected to ChIP with an antibody against MeCP2. Immunoprecipitated DNA was quantified by qRT-PCR with primers spanning the indicated pre-miRNA sequences. Data were normalized to input DNA. **d** Relative pri-miRNA expression levels of methylated and unmethylated miRNAs from WT mouse ESCs upon treatment with siMeCP2 or siControl. Data were normalized to *RPLP0*. **e** Relative levels of methylated and unmethylated mature miRNAs from WT mouse ESCs upon treatment with siMeCP2 or siControl. Data were normalized to the U6. All error bars represent ± SEM (n=3). * represents $p < 0.05$; ** represents $p < 0.01$; *** represents $p < 0.001$; NS = Not Significant; t-test. Source data are provided as a Source Data file.

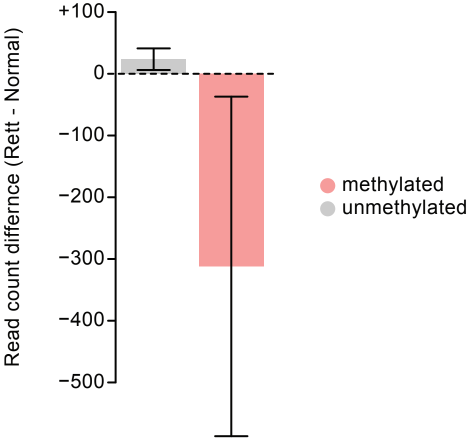

Supplementary Figure 6

Supplementary Fig. 6 MeCP2 binding to methylated miRNA loci slows Pol II elongation. The mean difference in read counts between RTT and normal mouse cerebellum plotted for methylated and unmethylated miRNAs.
