# Supplementary material for: DNA methylation directs microRNA biogenesis in mammalian cells
Source: Nat Commun. 2019 Dec 11;10:5657. doi: 10.1038/s41467-019-13527-1 (PMC6906426; doi:10.1038/s41467-019-13527-1)
Supplement: Supplementary file 2 — Reporting Summary [file 41467_2019_13527_MOESM2_ESM.pdf]

nature research

Corresponding author(s): Gil Ast and Carmit Levy

Last updated by author(s): Sep 20, 2019

Reporting Summary

Nature Research wishes to improve the reproducibility of the work that we publish. This form provides structure for consistency and transparency in reporting. For further information on Nature Research policies, see Authors & Referees and the Editorial Policy Checklist .

Statistics

For all statistical analyses, confirm that the following items are present in the figure legend, table legend, main text, or Methods section.

n/a	Confirmed	
☐	☒	The exact sample size (*n*) for each experimental group/condition, given as a discrete number and unit of measurement
☐	☒	A statement on whether measurements were taken from distinct samples or whether the same sample was measured repeatedly
☐	☒	The statistical test(s) used AND whether they are one- or two-sided<br>*Only common tests should be described solely by name; describe more complex techniques in the Methods section.*
☐	☒	A description of all covariates tested
☐	☒	A description of any assumptions or corrections, such as tests of normality and adjustment for multiple comparisons
☐	☒	A full description of the statistical parameters including central tendency (e.g. means) or other basic estimates (e.g. regression coefficient) AND variation (e.g. standard deviation) or associated estimates of uncertainty (e.g. confidence intervals)
☐	☒	For null hypothesis testing, the test statistic (e.g. *F*, *t*, *r*) with confidence intervals, effect sizes, degrees of freedom and *P* value noted<br>*Give P values as exact values whenever suitable.*
☐	☒	For Bayesian analysis, information on the choice of priors and Markov chain Monte Carlo settings
☐	☒	For hierarchical and complex designs, identification of the appropriate level for tests and full reporting of outcomes
☐	☒	Estimates of effect sizes (e.g. Cohen's *d*, Pearson's *r*), indicating how they were calculated

Our web collection on statistics for biologists contains articles on many of the points above.

Software and code

Policy information about availability of computer code

Data collection

Genomic coordinates of miRNAs
Genomic coordinates of human (GRCh37/hg19) and mouse (GRCm38/mm10) miRNAs were downloaded from miRBase65 (miRBase v19, http://www.mirbase.org/).
Additional datasets
Conservations scores for both human and mouse were downloaded from UCSC, mm10.60way.phastCons for mouse and hg19.100way.phastCons for human. The MNase-seq data from mouse R1 and human ESC were taken from the GEO (GSE64910 and GSE76083, respectively). ChIP-seq datasets for human HCT116 cells were taken from the GEO (MeCP2, GSM1154509; Pol II p-Ser2, GSE47677; and SP1, GSM1010902). ChIP-seq of total Pol II in mouse WT cells was downloaded from the GEO (GSM1446977). We downloaded raw reads and mapped them to the mouse (GRCm38/mm10) and human (GRCh37/hg19) reference genomes using bowtie267. Reads with mapping quality < 30 were discarded using samtools (http://www.htslib.org/). We used bam2wig.pl (http://search.cpan.org/~tjparnell/Bio-ToolBox-1.44/) to normalize base coverage to the total number of mapped reads and to construct a standard UCSC BigWig file. We used bwtool68 to extract values from BigWig files into BED files with coordinates. For ChIP-seq we subtracted the input signal from the ChIP-seq signal and for MNase-seq we extended each read to the length of 147 bp. BigWig files for GC and CpG content for both human and mouse were created using in-house Perl scripts.

Data analysis

Methylation profiles for mouse and human were taken from the ENCODE project. We used whole genome bisulfite sequencing (WGBS) data from postnatal 0 day mouse intestine (ENCSR353IFP), heart (ENCSR397YEG), kidney (ENCSR128HOP), liver (ENCSR550CYA), and lung (ENCSR409HKJ). Raw reads of WGBS from mouse embryonic stem cells were downloaded from the GEO (GSE82125) and were processed using Bismark66. For humans, we used WGBS data from ENCODE. We downloaded data for brain (ENCSR145HNT), heart (ENCSR699ETV), liver (ENCSR351IPU), lung (ENCSR797TEV), skin (ENCSR128RMY), intestine (ENCSR522UKJ), and embryonic stem cells (ENCSR617FKV). The DNA methylation patterns flanking the pre-miRNAs regions were used to define the methylated groups and the unmethylated groups. The methylated miRNAs were identified as miRNAs with high mean methylation levels 25 bp upstream of the pre-miRNA start positions and 25 bp downstream of the pre-miRNA end positions. The depleted miRNAs were defined as having no methylation within the pre-miRNA regions and 250 bp upstream or downstream. The rest of the miRNAs constitute the flat group. The same algorithm was

used to identify methylated and unmethylated miRNAs in mouse and in human cell lines.

For manuscripts utilizing custom algorithms or software that are central to the research but not yet described in published literature, software must be made available to editors/reviewers. We strongly encourage code deposition in a community repository (e.g. GitHub). See the Nature Research guidelines for submitting code & software for further information.

Data

Policy information about availability of data

All manuscripts must include a data availability statement. This statement should provide the following information, where applicable:

- Accession codes, unique identifiers, or web links for publicly available datasets
- A list of figures that have associated raw data
- A description of any restrictions on data availability

Provide your data availability statement here.

Field-specific reporting

Please select the one below that is the best fit for your research. If you are not sure, read the appropriate sections before making your selection.

[x] Life sciences [ ] Behavioural & social sciences [ ] Ecological, evolutionary & environmental sciences

For a reference copy of the document with all sections, see nature.com/documents/nr-reporting-summary-flat.pdf

Life sciences study design

All studies must disclose on these points even when the disclosure is negative.

Sample size	Not relevant. For statistics analysis we used t-test only for experimental data.
Data exclusions	No data was excluded from the analysis.
Replication	Not relevant.
Randomization	Not relevant.
Blinding	Not relevant.

Reporting for specific materials, systems and methods

We require information from authors about some types of materials, experimental systems and methods used in many studies. Here, indicate whether each material, system or method listed is relevant to your study. If you are not sure if a list item applies to your research, read the appropriate section before selecting a response.

Materials & experimental systems

n/a	Involved in the study	
[ ]	[x]	Antibodies
[ ]	[x]	Eukaryotic cell lines
[x]	[ ]	Palaeontology
[x]	[ ]	Animals and other organisms
[x]	[ ]	Human research participants
[x]	[ ]	Clinical data

Methods

n/a	Involved in the study	
[x]	[ ]	ChIP-seq
[x]	[ ]	Flow cytometry
[x]	[ ]	MRI-based neuroimaging

Antibodies

Antibodies used	anti-Drosha (Proteintech, 55001-1-AP), anti-GAPDH (GenScript, A00191-40), normal rabbit IgG (Santa Cruz Biotechnology, sc-2027), anti-H3K36me3 (Abcam, ab9050), anti-Pol II pSer2 (Abcam, ab5095), anti-Drosha (Abcam, ab12286), anti-Drosha (Santa Cruz Biothecnology, sc-31159), anti-RNA polymerase II CTD repeat YSPTSPS pSer5 (Abcam, ab5408), anti-RNA polymerase II CTD repeat YSPTSPS pSer2 (Abcam, ab5095, Abcam), and anti-MeCP2 (Cell Signaling, 3456)
Validation	Not relevant.

Eukaryotic cell lines

Policy information about cell lines

Cell line source(s)	WT R1 cells and TKO cells were a gift from Eran Meshorer's lab at the Hebrew University. Generated by: Tsumura, A. et al. Maintenance of self-renewal ability of mouse embryonic stem cells in the absence of DNA methyltransferases Dnmt1, Dnmt3a and Dnmt3b. Genes Cells 11, 805-814, doi:10.1111/j.1365-2443.2006.00984.x (2006).
Authentication	Cell lines were authenticated using standard PCR and standard western blotting.
Mycoplasma contamination	All cells were tested negative for Mycoplasma contamination.
Commonly misidentified lines (See ICLAC register)	Not relevant.
