# Supplementary material for: DNA methylation directs microRNA biogenesis in mammalian cells
Source: Nat Commun. 2019 Dec 11;10:5657. doi: 10.1038/s41467-019-13527-1 (PMC6906426; doi:10.1038/s41467-019-13527-1)
Supplement: Supplementary file 3 — Description of Additional Supplementary Files [file 41467_2019_13527_MOESM3_ESM.docx]

**Description of Additional Supplementary Files**

File name: Supplementary Data 1
Description: List of miRNAs from each sub-group from human and mouse

File name: Supplementary Data 2
Description: KEGG analyses of methylated and unmethylated miRNAs

File name: Supplementary Data 3
Description: siRNA oligonucleotide sequences and primer sequences that were used in our study
